# Nitrogen Fertilizer Application Alters the Root Endophyte Bacterial Microbiome in Maize Plants, but Not in the Stem or Rhizosphere Soil

**DOI:** 10.1128/spectrum.01785-22

**Published:** 2022-10-18

**Authors:** Alejandra Miranda-Carrazco, Yendi E. Navarro-Noya, Bram Govaerts, Nele Verhulst, Luc Dendooven

**Affiliations:** a Laboratory of Soil Ecology, Cinvestav, Mexico City, Mexico; b Centro de Investigación en Ciencias Biológicas, Universidad Autónoma de Tlaxcala, Tlaxcala, México; c International Maize and Wheat Improvement Centre (CIMMYT) Mexico, Mexico City, Mexico; d Cornell University, Ithaca, New York, USA; Yeungnam University

**Keywords:** agricultural practices, bacterial community structure, DArT-seq, functionality of maize bacterial community, genes involved in N cycling

## Abstract

Plant-associated microorganisms that affect plant development, their composition, and their functionality are determined by the host, soil conditions, and agricultural practices. How agricultural practices affect the rhizosphere microbiome has been well studied, but less is known about how they might affect plant endophytes. In this study, the metagenomic DNA from the rhizosphere and endophyte communities of root and stem of maize plants was extracted and sequenced with the “diversity arrays technology sequencing,” while the bacterial community and functionality (organized by subsystems from general to specific functions) were investigated in crops cultivated with or without tillage and with or without N fertilizer application. Tillage had a small significant effect on the bacterial community in the rhizosphere, but N fertilizer had a highly significant effect on the roots, but not on the rhizosphere or stem. The relative abundance of many bacterial species was significantly different in the roots and stem of fertilized maize plants, but not in the unfertilized ones. The abundance of N cycle genes was affected by N fertilization application, most accentuated in the roots. How these changes in bacterial composition and N genes composition might affect plant development or crop yields has still to be unraveled.

**IMPORTANCE** We investigated the bacterial community structure in the rhizosphere, root, and stem of maize plants cultivated under different agricultural techniques, i.e., with or without N fertilization, and with or without tillage. We found that the bacterial community was defined mostly by the plant compartment and less by agricultural techniques. In the roots, N fertilizer application affected the bacterial community structure, the microbiome functionality, and the abundance of genes involved in the N cycle, but the effect in the rhizosphere and stem was much smaller. Contrary, tillage did not affect the maize microbiome. This study enriches our knowledge about the plant-microbiome system and how N fertilization application affected it.

## INTRODUCTION

Plants live in association with a large variety of microorganisms, which have a huge effect on their development and fitness. A plant and its associated microbiota or endophytes can be considered a single entity, the holobiont (1, 2). A better understanding of the plant-associated microorganisms and their functionality is one of the most important challenges in agriculture to improve yields in a sustainable way.

The relationship between a plant and its endophytes can be mutualistic, beneficial, commensal, or neutral but also pathogenic (3). Plant-associated microbiota have been shown to promote plant growth, nutrient acquisition, disease resistance, and stress tolerance (2). The structure and diversity of the plant microbiome are determined by the host, i.e., plant species, genotype, age, immunity, and the compartment, the microorganisms and environmental factors, such as soil characteristics, cultivation practices, and climatic conditions (4). The rhizosphere microbiome formed from the interaction of the plant with the soil microorganisms becomes an important source of plant endophytes (5). However, microorganisms found on and embedded in the seeds will also contribute to the plant biome (6).

In 1992, the “Centro Internacional de Mejoramiento de Maíz y Trigo” (CIMMYT) started a long-term field trial at “Campo Experimental Norman E. Borlaug (CENEB)” in the Yaqui Valley in the northwestern part of Mexico to study the effect of different agricultural practices, i.e., residue management, tillage, and different N fertilization application rates, on soil characteristics and crop yields (7). The field trial had a maize (Zea mays L.) and wheat (*Triticum durum* L.) crop rotation. In the Yaqui Valley, farmers mainly grow durum wheat in monoculture in the winter under furrow irrigation and sometimes maize as a summer crop. Maize, grown for its grain and biomass, is one of the most important cereals in the world and is used as raw material in many industries. Maize is cultivated in different environments characterized by a wide range of climates, i.e., rainfall, temperatures, altitudes, and soil types (8). The agricultural techniques used to cultivate maize, e.g., bed planting, inorganic N fertilizer application, and tillage, have changed through time to improve grain quality and crop yield. How these agricultural practices affect crop yields has been studied intensively, as also how they may alter the soil microbial community (9–11). Less is known, however, how these agricultural practices, i.e., inorganic N fertilizer application and tillage, might affect the plant endophytes.

Therefore, four different treatments from the experimental trial at CENEB were included in this study. They combined two N fertilizer application rates, i.e., no fertilizer versus 300 kg urea-N ha^−1^, and two tillage-residue management practices, i.e., tilled bed planting with residue incorporated and permanent beds with residue retained. Earlier research showed that retention of crop residue reduced N availability affecting C and N dynamics, grain quality, and the bacterial communities (12–14). The aim of this study was to investigate how two agricultural practices, i.e., tillage and N fertilizer application, affected the bacterial community structure and the microbial functionality in the rhizosphere, roots, and stem of irrigated maize plants cultivated in a Vertisol soil. The DNA from rhizosphere and root and stem endophytes was extracted and then sequenced through Diversity Arrays Technology sequencing (DArT-seq). Taxonomic and functional annotations were obtained with bioinformatics tools and submitted to statistics to compare communities from different plant compartments and agricultural practices.

## RESULTS

### Sequencing analysis.

After the quality filtering and the removal of sequences aligned with maize and human reference genomes, a total of 135,794,384 sequences were obtained from 36 samples, representing 18 bacterial phyla, 760 genera, and 2,388 species. Overall, 31 functional traits were defined at subsystem 1, 104 at subsystem 2, 532 at subsystem 3, and 1,824 at all function levels (Table S1).

### Effect of agricultural practices on the bacterial community.

**FIG 1:**
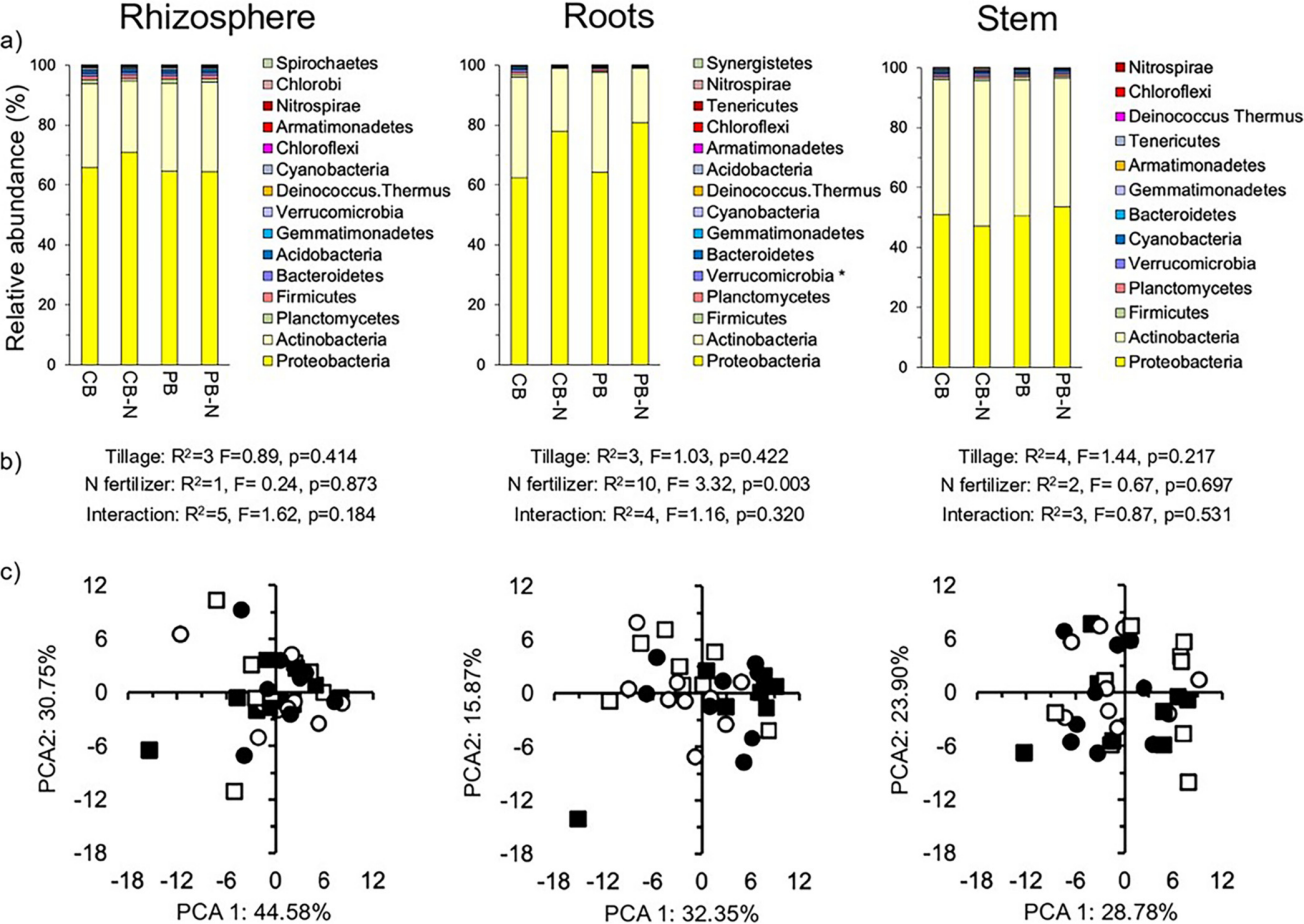
Bacterial community at phylum level. (a) Relative abundance (%) of the most abundant bacterial phyla in the rhizosphere, roots, and stem of maize (Zea mays L.) in soil with conventional tilled beds left unfertilized (CB) or fertilized (CB-N) and permanent beds left unfertilized (PB) or fertilized (PB-N). (b) The effect of tillage, N fertilizer application, and their interaction on the bacterial community structure, considering all bacterial phyla determined with the permutational multivariate analyses of variance (perMANOVA) test. (c) A principal-component analysis (PCA) with all bacterial phyla in the CB (□), CB-N (■), PB (○), and PB-N (●).

Proteobacteria and Actinobacteria were the dominant bacterial phyla in the rhizosphere, roots, and stem of the maize plant and accounted for >94% of all bacterial groups (Fig. 1a). None of the bacterial phyla were significantly affected by N fertilizer application or tillage in the rhizosphere, roots, or stem of the maize plants when considering the bacterial phyla separately (Fig. S2a). The effect size of none of the bacterial phyla was large when comparing the fertilized versus the unfertilized plants except for the Verrucomicrobia (effect size −0.9), i.e., more abundant in the roots of unfertilized maize plants than in the fertilized plants, and Proteobacteria (effect size 0.9), i.e., was more abundant in the roots of fertilized maize plants than in the unfertilized ones (Fig. 1a, Fig. S2a). Nitrogen fertilizer application had a significant effect on the whole bacterial community structure considering all bacterial phyla in the roots (*P* = 0.003, R^2^ = 10), but not in the rhizosphere or stem (Fig. 1b and c).

**FIG 2:**
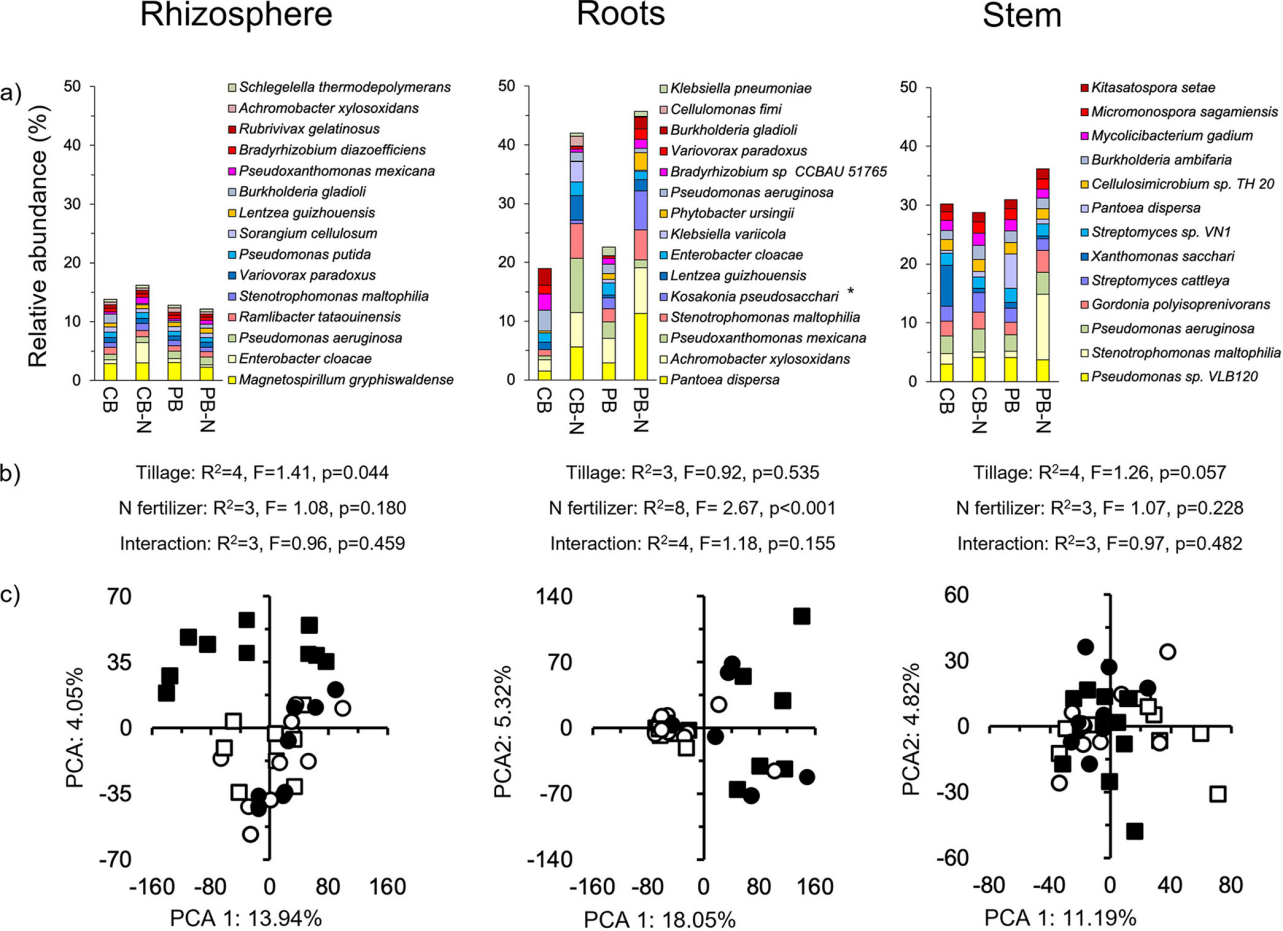
(a) Relative abundance (%) of the most abundant bacterial species in the rhizosphere, roots, and stem of maize (Zea mays L.) in soil with conventional tilled beds left unfertilized (CB) or fertilized (CB-N) and permanent beds left unfertilized (PB) or fertilized (PB-N). (b) The effect of tillage, N fertilizer application, and their interaction on the bacterial community structure, considering all bacterial species determined with the permutational multivariate analyses of variance (perMANOVA) test. (c) A principal-component analysis (PCA) with all bacterial phyla in the CB (□), CB-N (■), PB (○), and PB-N (●).

Tillage had no significant effect on the alpha diversity (qDα) based on Hill equivalent numbers in the rhizosphere, roots, and stem of the maize plants (Fig. S3a). Application of N fertilization, however, had a significant effect on the alpha diversity (qDα) considering *q *= 0 (*P* = 0.009) in the roots of the maize plants, but not in the rhizosphere or stem, or considering *q *= 1 or *q *= 2. Magnetospirillum gryphiswaldense was the most abundant bacterial species in the rhizosphere (2.81±0.35%), Pantoea dispersa in the root (5.33±4.33%), and Pseudomonas sp. *VLB120* in the stem (3.75±0.54%) (Fig. 2a). The effect size when comparing the relative abundance of bacterial species in the unfertilized versus the fertilized maize plants was sometimes very large (≥1.3 or ≤−1.3), affecting more bacterial species in the roots of the maize plants than in the stem or rhizosphere (Fig. S2b, Table S2). Tillage had a small significant effect on the bacterial community in the rhizosphere (*P* = 0.044), but not in the stem or roots of the maize plants (Fig. 2b). Nitrogen fertilizer application had a highly significant effect on the bacterial community in the roots (*P* < 0.001, R^2^ = 8), but not in the rhizosphere or stem. Although the effect of N fertilizer application was highly significant, the different treatments were not clearly separated in the PCA as only 10% of the variation (R^2^) was explained by it (Fig. 2c).

### Effect of maize plant compartment on the bacterial community.

**FIG 3:**
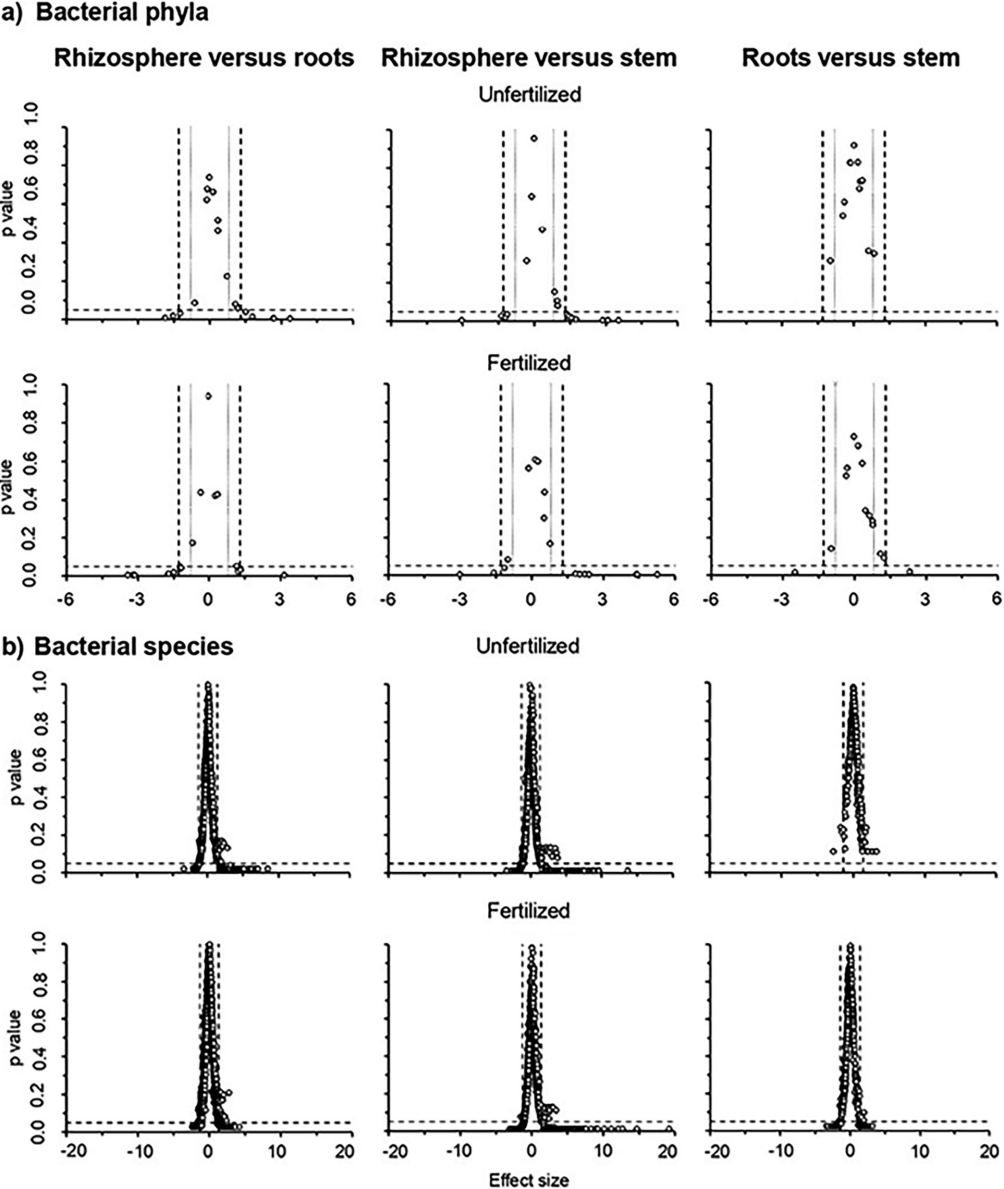
Volcano plot comparing the relative abundance of bacterial (a) phyla and (b) species in the rhizosphere, roots, and stem of maize plants (Zea mays L.) in the fertilized and unfertilized soil with the effect size in the *x* axis and the expected value of the Benjamini-Hochberg corrected *P*-value in the *y* axis. The expected value of the Benjamini-Hochberg corrected *P*-value and the effect size, which is defined as the difference between groups divided by the maximum dispersion within group A or B, was calculated with the ALDEx2 package using the aldex.ttest argument (52).

**FIG 4:**
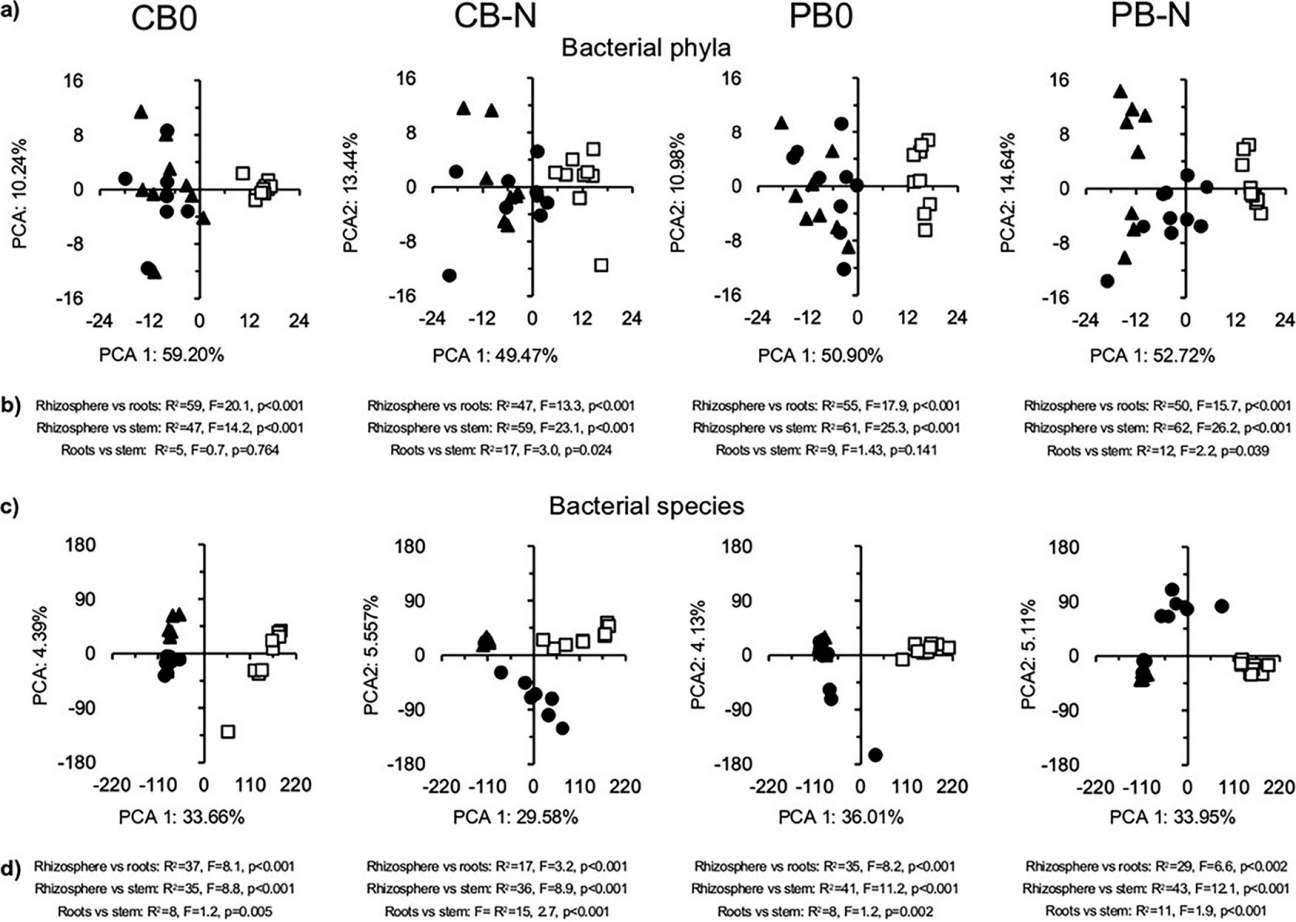
(a) Principal-component analysis (PCA) with (a) all bacterial phyla in the rhizosphere (□), roots (●), and stem of maize (Zea mays L.) (□) cultivated in soil with conventional tilled beds left unfertilized (CB) or fertilized (CB-N) and permanent beds left unfertilized (PB) or fertilized (PB-N). (b) Permutational multivariate analyses of variance (perMANOVA) test comparing the bacterial community structure in the rhizosphere versus the roots and stem, and comparing the roots with the stem, considering all bacterial phyla. (c) PCA with all bacterial species. (d) PerMANOVA test comparing the bacterial community structure, considering all bacterial species in the rhizosphere versus the roots and stem, and comparing the roots with the stem.

The relative abundance of a large number of bacterial phyla in the rhizosphere was significantly different from that in the roots and stem of the maize plant with an effect size often ≥1.3 or ≤−1.3 most accentuated in the fertilized soil (Fig. 3a). For instance, the relative abundance of members of the Acidobacteria was significantly higher in the roots of N fertilized maize plants than in the stem (*P* = 0.016, effect size 2.5), and that of Proteobacteria showed an opposite effect (*P* = 0.019, effect size 2.3), but none was seen in the unfertilized soil (Table S3). The PCA clearly separated the bacterial community structure in the rhizosphere from that in the roots and stem of the maize plant considering all bacterial phyla independent of the agriculture practices applied (Fig. 4a). Consequently, the bacterial community structure in the rhizosphere considering all bacterial phyla was highly significantly different from that of the maize plant endophytes independent from the agriculture practices applied (*P* < 0.001) (Fig. 4b). The bacterial community structure in the roots was also significantly different from that in the stem in the fertilized soil, considering all bacterial phyla (*P* < 0.05), but not in the unfertilized soil.

The alpha diversity (qDα) based on Hill equivalent numbers at *q *= 0, *q *= 1, and *q *= 2 of the bacterial species was significantly higher in the rhizosphere than in the plant stem or roots (*P* < 0.001) (Fig. S3a). The relative abundance of many bacterial species in the rhizosphere was significantly different from that in the roots and stem of the maize plant with an effect size often very large (≥1.3 or ≤ −1.3) (Fig. 3b, Table S4). The relative abundance of many bacterial species in the roots was also significantly different from that in the stem of the fertilized maize plant (*P* < 0.001), but none in the unfertilized plants, although the effect size was often very large (≥1.3 or ≤ −1.3). The PCA clearly separated the bacterial community structure in the rhizosphere from that in the roots and stem of the maize plants, considering all bacterial species independent of the agriculture practices applied (Fig. 4c), and the bacterial community structure was highly significantly different between the rhizosphere, roots, and stem of the maize plants (p ≤ 0.005) (Fig. 4d).

### Functional annotation of plant-associated whole communities.

The alpha diversity (qDα) based on Hill equivalent numbers at *q *= 0, *q *= 1, and *q *= 2 of the functional annotation was not affected by tillage in the rhizosphere, roots, or stem of the maize plants, while N fertilizer had a significant effect on *q *= 1 in the stem and on *q *= 2 in the roots (*P* < 0.05) (Fig. S3b). Diversity at *q *= 0, *q *= 1, and *q *= 2 was significantly higher in the rhizosphere than in the roots and stem of maize plants and at *q *= 1 also significantly higher in the roots than in the stem when maize plants were cultivated on conventional tilled beds. In the unfertilized soil, diversity at *q *= 0, *q *= 1 and *q *= 2 was significantly higher in the rhizosphere than in the roots and stem of maize plants (*P* < 0.05). In the fertilized soil, however, the diversity at *q *= 1 and *q *= 2 was significantly higher in the roots than in the stem of the maize plants, but significantly lower than in the rhizosphere (*P* < 0.05). At q = 0 in the fertilized soil, the diversity was only significantly higher in the rhizosphere than in the stem.

**FIG 5:**
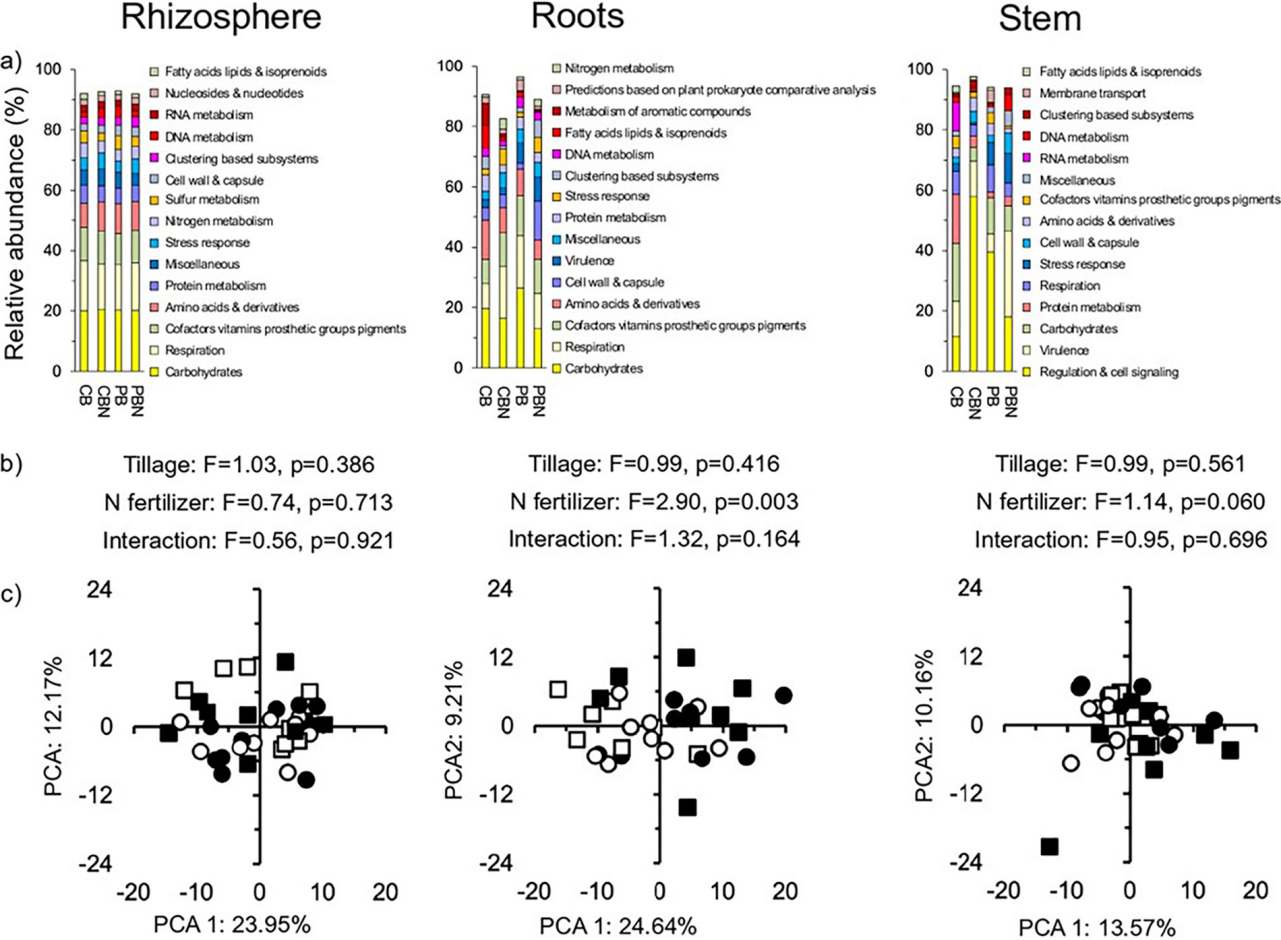
(a) Relative abundance (%) of the most abundant bacterial traits at level 1 in the rhizosphere, roots, and stem of maize (Zea mays L.) in soil with conventional tilled beds left unfertilized (CB) or fertilized (CB-N) and permanent beds left unfertilized (PB) or fertilized (PB-N). (b) Permutational multivariate analyses of variance (perMANOVA) test to determine the effect of tillage, N fertilizer application, and their interaction on the bacterial traits at level 3. (c) Principal-component analysis (PCA) with all bacterial traits at level 3 in the CB (□), CB-N (■), PB (○), and PB-N (●).

At level 1 of functionality, carbohydrates metabolism was the most abundant functional trait of the bacteria in the rhizosphere and roots, and regulation and cell signaling in the stem (Fig. 5a). At level 3 of functionality, the respiratory complex I was the most abundant functional trait in the rhizosphere (10.04±3.57%), copper homeostasis in the roots (9.93±19.04%), and phosphoinositides biosynthesis (26.21±29.26%) in the stem (Fig. S4a). The relative abundance of none of the functional traits at level 1 (data not shown) or level 3 when comparing the effect of tillage or N fertilizer application was significant or the effect size was large (≥0.8 or ≤−0.8) in the rhizosphere, roots, or stem of the maize plant (Fig. S5a). Nitrogen fertilizer application had a highly significant effect on the functional traits in the roots, considering level 1, and in the roots and stem, considering level 3 (*P* ≤ 0.009) (Fig. 5b, Fig. S4b, S4c).

In the unfertilized and fertilized soil, the relative abundance of the functional traits at level 3 was not significantly different in the rhizosphere from that in the roots or stem, and between the stem and the roots (Fig. S5b). The effect size, however, was sometimes large (≥0.8 or ≤−0.8) when comparing the relative abundance of the functional traits at level 3 in the rhizosphere and the roots and very large (≥1.3 or ≤−1.3) when comparing the rhizosphere with the stem in the unfertilized and fertilized soil. When comparing the relative abundance of the functional traits at level 3 in the roots with that in the stem, however, the effect size, was only large (≥0.8 or ≤−0.8) for some traits in the fertilized soil. The functional traits at level 1 and 3 in the rhizosphere were significantly different from those in the stem and roots of the maize plants, and the PCA separated them clearly (*P* < 0.05) (Fig. S6). The functional traits were only highly significant different between the stem and the roots, considering level 1 in the fertilized maize plants (*P* ≤ 0.004) (Fig. S6b).

### Genes involved in the N cycle.

**FIG 6:**
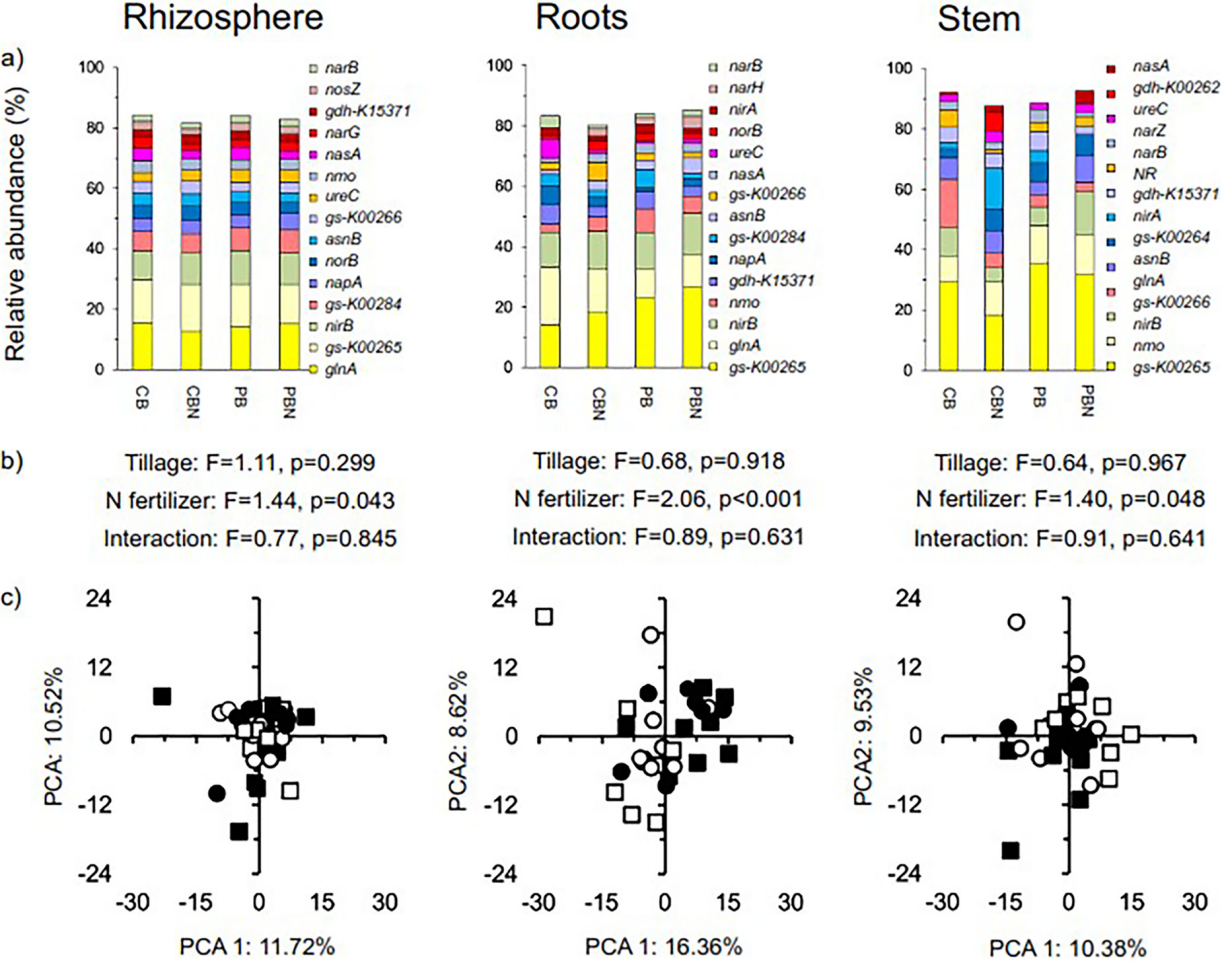
(a) Relative abundance (%) of the most abundant genes involved in the N cycle in the rhizosphere, roots, and stem of maize (Zea mays L.) in soil with conventional tilled beds left unfertilized (CB) or fertilized (CB-N) and permanent beds left unfertilized (PB) or fertilized (PB-N). (b) Effect of tillage, N fertilizer application, and their interaction on the genes involved in the N cycle determined with the permutational multivariate analyses of variance (perMANOVA) test. (c) Principal-component analysis (PCA) with all genes involved in the N cycle in the CB (□), CB-N (■), PB (○), and PB-N (●).

Genes that encoded glutamate synthases that transform 2 l-glutamate to l-glutamine and that participate with glutamine synthetase in the ammonia assimilation processes were the most abundant in the rhizosphere, roots, and stem of the maize plants (Fig. 6a). Genes that encoded nitrite reductase, glutamine synthetases, and nitrate reductase were the next most abundant. Application of N fertilizer or tillage had no significant effect on individual genes involved in N cycling or when grouped per process, and the effect size was never large (<0.8 and >−0.8) (Fig. S7a).

Tillage practice had no significant effect on different genes involved in N cycling, but the effect size when comparing those in the rhizosphere with those in the roots or stem, and those in the roots with those in the stem, were often large (≥0.8 or ≤−0.8), more accentuated in the unfertilized than in the fertilized plants (Fig. S7b). The PCA clearly separated the relative abundance of genes involved in N cycling from those in the roots and stem of the maize plants, and the effect was highly significant (*P* < 0.001) (Fig. S8). The abundance of genes that coded for nitronate monooxygenase was significantly higher in the stem than in the rhizosphere (*P* = 0.007), while the abundance of genes that coded for reductases involved in the denitrification process (sum of all genes) was higher in the rhizosphere than in the stem of the maize plants (*P* = 0.028). Nitrogen fertilizer application had a significant effect on the compositional structure of the genes involved in the N cycle in the rhizosphere and stem of maize (*P* < 0.05) and a highly significant effect on the roots of the maize plants (*P* < 0.001) (Fig. 6b). The relative abundance of the genes involved in the N cycle was highly significantly different between the rhizosphere and the roots or stem independently of the agricultural practices applied (*P* < 0.001). The relative abundance of genes involved in the N cycle was also highly significantly different between the stem and the roots in the soil fertilized with 300 kg N ha^−1^ y^−1^ (*P* < 0.001). The PCA did not separate the different agricultural practices in the rhizosphere and stem of the maize plants, considering the genes involved in the N cycle, although it did in the roots (Fig. 6c).

## DISCUSSION

Plant endophytes play an important role in N and P cycling, protecting their host against pathogens, and might have plant-growth promotion capabilities (15). They might originate in the seeds or enter the plant via the roots or leaves (6, 16, 17). The microorganisms that enter a plant from the rhizosphere will be mostly defined by the characteristics of the plant and the potential endophytes. However, soil conditions are known to affect the rhizosphere microorganisms and plant development, so it can be hypothesized that factors that change them might also alter the plant biome. In this study, we investigated how two agricultural practices, i.e., tillage and N fertilizer application, affected the bacterial community structure and microbial functionality in the rhizosphere, roots, and stem of maize plants.

### Effect of tillage.

Tillage had only a small effect on the bacterial community structure, functionality, and genes involved in the N cycle. It had a small significant effect on the bacterial community, considering the bacterial species in the rhizosphere, but not in the roots or stem and had no significant effect on the alpha diversity, bacterial functionality, or genes involved in the N cycle in the rhizosphere, roots, or stem. The relative abundance of only five bacterial species had an effect size that could be considered very large when comparing bacterial communities in the conventional tilled beds with those in permanent tilled beds. The relative abundance of Enterobacter hormaechei with P and K solubilizing capacities (18) and *Pontibacter pudoricolor* (19) was larger in the rhizosphere of maize plants cultivated on CB than in PB, while that of the Actinobacteria Conexibacter woesei (20) and *Mycolicibacterium tokaiense* (21) was larger in roots when the maize plants were cultivated on PB than on CB. The relative abundance of *Rhodococcus* sp. PBTS 1 that causes pistachio bushy top syndrome (PBTS), a recently emerged disease that has strongly impacted the pistachio industry (22), was lower in the stem of maize plants cultivated on PB than on CB. Most of these bacteria have been described only recently while their metabolic characteristics are still largely unknown.

### Effect of N fertilizer application.

N fertilizer application had a larger effect on the bacterial community structure, the functionality, and genes involved in the N cycle in the roots of the maize plants than tillage, but the effect on the rhizosphere and stem of maize plants was similar. For instance, the relative abundance of 12 bacterial species was higher in the roots when N fertilizer was applied than when it was not, but only three were seen in the rhizosphere, none in the stem, and eight when no N fertilizer was applied, compared to the fertilized roots but only three in the rhizosphere and one in the stem. This did, however, not necessarily mean that the bacterial species were enriched in the roots compared to the rhizosphere, but mostly that the increase or decrease in relative abundance was larger or smaller in the first than in the latter, i.e., N fertilizer application increased the relative abundance or limited its decrease compared to the unfertilized plants. For instance, the relative abundance of *Mesorhizobium terrae* (recently isolated from a soil in South Korea [23]) decreased in both the unfertilized and fertilized soil plant roots but the decrease was lower in the fertilized than in the unfertilized soil. The relative abundance of only two bacterial species decreased in the roots of the unfertilized plant versus the rhizosphere soil and increased in the fertilized plant roots versus the rhizosphere soil, i.e., Cellulomonas fimi and *Stenotrophomonas sp SAU14A_NAIMI4_5* that belongs to the S. maltophilia complex (24). Cellulomonas fimi secretes complex mixtures of carbohydrate active enzymes (CAZymes) that degrade synergistically hemicelluloses and cellulose (25). Members of the species Stenotrophomonas maltophilia participate in the sulfur and nitrogen cycles, degrade complex pollutants, promote plant growth, and are found in different environmental habitats although they are associated mainly with plants (26). Stenotrophomonas maltophilia has also emerged as a global opportunistic human pathogen (27).

In the rhizosphere, the relative abundance of two ammonia-oxidizing bacteria, Nitrosomonas communis and *Nitrospira multiformis,* was higher when fertilized with N than when left unfertilized. In the N fertilized soil, hydrolysis of the applied urea will provide ammonium oxidizers with an energy source, which will be less available in the unfertilized soil so that their relative abundance will be larger in the first than in the latter. The relative abundance of Mycobacterium intracellulare increased in the stem of the maize plants, but the increase was larger in the N fertilized soil than in the unfertilized soil. Mycobacterium intracellulare is part of the Mycobacterium avium complex (28) and part of the nontuberculous mycobacteria found in soil and water (29). Why Mycobacterium intracellulare would be enriched in the stem of maize plants is difficult to determine, although they are ubiquitous in soil (30).

Although the bacterial functionality structure at level 3 was significantly different between the unfertilized and N fertilized plots, none of the specific functionalities was different. The bacterial population in the maize plant is very diverse, but it appears that their functionality or metabolic diversity is less so. This might suggest that although N fertilizer application had a large effect on specific bacterial species the effect on specific functions was smaller. As such, N fertilizer application favors certain metabolic functions that a wide range of bacteria in the maize plant might possess.

It should be remembered, however, that the plant biomass, N content, and yields will be higher in the 300 kg ha-1 fertilized soil than in the unfertilized soil. For instance, Graham et al. (2014) reported that in the same experiment yields of durum wheat (*Triticum durum* L.) in the 300 kg ha-1 fertilized soil were nearly three times as high as those in the unfertilized soil, i.e., 3 ton ha^−1^ versus 8 ton ha^−1^ (12). These differences in plant development, biomass, and N deficiency in the crop between the unfertilized and 300 kg ha-1 fertilized crop might also affect microbial communities. It might be difficult, however, to separate the effect of reduced N availability *per se* from other factors that might be different between the fertilized and unfertilized crops, e.g., water content, other plant nutrients (micronutrients, P and K), or disease.

### The effect of the maize plant compartment.

The effect of the plant compartments on the bacterial community structure, functionality, and genes involved in the N cycle was greater than that of the agricultural practices. The plant-associated prokaryotes communities were affected by plant tissue; i.e., they were different between the roots and the stem (2, 4). As the different plant compartments provide different ecological niches, the endophytic community composition changed within the plant (4, 5, 31). The bacterial and functional diversity and richness declined from rhizosphere to stem, which suggests a stronger competition among microorganisms in the aerial space of the plant as the habitat is more tightly defined (5). Despite that, the same bacterial phyla, i.e., Proteobacteria and Actinobacteria, dominated in the rhizosphere, roots, and stem, while *Streptomyces* was the dominant genus except in the fertilized roots.

The bacterial community structure, the functionality, and the genes involved in the N cycle were clearly different between the rhizosphere, roots, and the stem. The effect was more accentuated in the N fertilized soil than in the unfertilized soil. This confirms that the bacterial community and its functionality are largely controlled by the plant, but some results indicate that an external factor such as N fertilizer application might affect this in the roots but not in the stem. A first possible explanation is that N fertilizer applications alter the relative abundance of certain bacteria in the rhizosphere so that more (or less) enter the roots; i.e., the abundance of the bacteria defines how many will enter the roots. However, this explanation was not supported by the results, since the relative abundance of the bacterial species that increased or decreased in the roots of N fertilized maize plants was similar in the rhizosphere. A second possible explanation is that N fertilizer application alters the barrier between the rhizosphere and the roots so that the number of bacteria that enter the roots is different, i.e., smaller, or larger, compared to the number that enter in the unfertilized roots. The latter explanation is supported by the data. For instance, the relative abundance of *Hydrogenophaga* sp BA0156 was similar in the rhizosphere (0.23%) of the N fertilized and unfertilized soil. Their relative abundance decreased in the roots of both the N fertilized and unfertilized soil, but the decrease in the first was smaller (relative abundance 0.08%) than in the latter (0.02%). This suggests that it might be difficult to use a bacterial biofertilizer (a single species or a bacterial consortium applied with or without an inorganic or organic carrier) to stimulate plant growth. It appears that only bacteria that can cross the soil plant barrier defined by soil conditions and/or plant characteristics might, if they have plant growth stimulating capacity, stimulate plant growth and ultimately crop yields. How soil characteristics such as texture and organic matter content, climatic conditions like rainfall and temperature, and plant characteristics, i.e., plant species, determine or control this soil (rhizosphere) plant barrier (roots) is still largely unknown, but it might be difficult to alter it without some genetic modification of the bacteria or plant, which in itself is a complex although extremely intriguing research topic with possible vast economic benefits if it can be achieved (32).

## CONCLUSION

The host compartment had a larger effect on the bacterial community structure and microbial functionality than agricultural practices. Tillage had a small significant effect on the bacterial community in the rhizosphere, but not in the stem or root of the maize plants. Nitrogen fertilizer application had a highly significant effect on the bacterial community structure and species richness in the roots, but not in the rhizosphere and stem. Tillage had no effect on the microbial functionality, but N fertilizer application had a highly significant effect on the functional traits in the roots, considering level 1, and in the roots and stem, considering level 3. The abundance of genes involved in the N cycle was significantly affected by N fertilization in the rhizosphere and stem, and highly significantly in the roots. How these changes in the root microbiome as a result of N fertilizer application might affect plant growth and crop yields has still to be investigated.

## MATERIALS AND METHODS

### Long-term trial and treatments description.

Rhizosphere soil and maize plants were collected from the long-term field experiment at Campo Experimental Norman E. Borlaug (CENEB), near Ciudad Obregón, Sonora, México (27.33 N, 109.09 W, 38 masl), started in 1992. According to the World Reference Base System, the soil is a Hyposodic Vertisol (Calcaric, Chromic) (33, 34) and the climate is BWh (hot desert climate system) Köppen–Geiger climate classification system (35).

The long-term experiment had a randomized complete block design with a split plot treatment arrangement and three replicated plots per treatment. Maize and wheat were grown in an annual rotation. Maize is planted in June and harvested in October. More details of the long-term trial can be found in Verhulst et al. (7). Sampled treatments included two tillage practices and two N fertilization application rates. A first treatment included conventionally tilled raised beds with no N fertilizer application; i.e., new beds were formed before each growing season with a disk harrow to 20 cm (considered the CB treatment); the second treatment included the same agricultural practices, but crops were fertilized with 300 kg urea N ha^−1^ (considered the CB-N treatment); a third treatment included permanent raised beds with zero tillage and continued reuse of existing beds with no N fertilizer applied (considered the PB treatment); and fourth a treatment included, the same permanent beds but fertilized with 300 kg urea-N ha^−1^ (considered the PB-N treatment). Crop residues were retained in the field in all treatments, incorporated by tillage in CB and CB-N and left at the soil surface in PB and PB-N. All treatments received 46 kg P_2_O_5_ ha^−1^ for wheat and 50 kg P_2_O_5_ ha^−1^ for maize, but no K was applied as the soil is rich in it. A furrow irrigation system is used at the experimental site. The climate is arid with a mean annual temperature of 24.7°C, and an average rainfall of 384 mm (14).

### Plant and soil sampling.

Three maize plants and their rhizosphere to a depth of 25 cm were taken from each replicated plot (*n *= 3) of each treatment (*n *= 4) (Fig. S1). As such, 36 plants and rhizospheres were collected. Plants and soil were transported in plastic bags from the CIMMYT’s experimental station in Obregon (Sonora) to the research center in Texcoco in Central Mexico and stored at −80°C. The field-based replication was maintained for DNA extraction to avoid pseudoreplication (36). Samples were taken in September 2019 when maize plants were in VT stage, i.e., tasseling.

### Tissue disinfestation.

Stems and roots of each plant were cut in small pieces and disinfested. Stems were washed with tap water (10 s), ethanol (70%) (2 min), distilled water (30 s), NaClO (Cl 2.5%) (5 min), and distilled water again (1 min each time) (37, 38). The sterility of the distilled water from the last step of disinfestation of each sample was tested spreading 50 μL in in a petri dish with LB medium. No bacteria grew in the media. As such, the washing method had no effect on the studied bacterial community and its functionality.

Roots were shaken for 10 min and the collected soil was considered the bulk soil. The roots were placed in 250 mL beaker added with NaCl 0.9% and shaken for 10 min. The soil retained in the NaCl solution was considered the rhizosphere soil and was saved in 15 mL Falcon tubes and extracted for DNA as described below. The roots were disinfested in the same way as the stems.

### Metagenomic DNA extraction.

Metagenomic DNA from roots and stems was extracted with the CTAB method (39). Briefly, the cells were lysed with CTAB at 65°C for 2 h and lipids, and polysaccharides and proteins were removed with chloroform:octanol 24:1. Nucleic acids were precipitated with isopropanol and purified with 70% ethanol. The DNA from rhizosphere soil was extracted with the Quick-DNA Fecal/Soil Microbe kit (Zymo Research, CA, USA). The DNA quality was determined by electrophoresis in 1% agarose gels in 1× TAE buffer and staining with SYBR Gold (Invitrogen, Carlsbad, CA, USA) and quantified with a NanoDrop 8000 spectrophotometer V 2.1.0 (Mettler Toledo, ZH, Switzerland). Controls, i.e., solutions used in the DNA extraction process, were included in each protocol to guarantee that the DNA obtained was not the result of a contamination of the reagents.

### DNA sequencing using the DArT-seq method.

The high-throughput genotyping method diversity arrays technologýs sequencing (DArT-seq) (40) was used to obtain the metagenomic DNA reads. The procedure was done at the DArT laboratory in Australia. The DNA samples were digested using a combination of two restriction enzymes, PstI and HpaII. A PstI-compatible adapter included the Illumina flowcell attachment sequence, the sequencing primer (AATGATACGGCGACCACCGAGATCTACACTCTTTCCCTACACGACGCTCTTCCGATCT), and various length barcode regions. The reverse adapter contained the flowcell attachment region and the HpaII compatible overhang sequence (CAAGCAGAAGACGGCATACGAGATCGGTCTCGGCATTCCTGCTGAACCGCTCTTCCGATCTCGG). Only fragments containing the PstI-HpaII end were amplified. Equimolar amounts of PCR products (100 ng μL^−1^) from each sample were purified and sequenced on a flow cell SP of 100 cycles on Illumina NovaSeq 6000 (Illumina Inc., CA, USA) (41).

### Bioinformatic analyses.

Sequences quality in the FASTQ format was checked with the FastQC software (42) and trimming using Trimmomatic tool (43). Sequences with low and medium quality, i.e., less than 28 Phred score, and primes (19 nt at the beginning of each sequence) were discarded. The trimmed reads were mapped to the maize (GCF_902167145.1) and human (GCF_000001405.39) representative genomes, which were downloaded from NCBI database (https://www.ncbi.nlm.nih.gov/refseq/), using bbmap software (44) to identify and remove the plant host originated reads and possible human contamination. The taxonomic annotations were obtained from read-based metagenomic analyses using the trimmed and filtered reads, i.e., clean reads, through Kraken2 v. 2.0.7 (45), and the abundance estimation was done with the argument estimate_abuncance.py with the Bracken tool (46) using a database with a kmer length of 63 nt. Data containing information of bacterial phylum, genus, and species were used. Clean reads were used as inputs for the functional analyses with the SUPER-FOCUS software (47) using RAPSearch2 aligner (48) against the SUPER-FOCUS database with 95% of sensitivity. SUPER-FOCUS uses a reduced database organized by a subsystem (three levels) structure of metagenomic data sets in which the first level is the most general class, while the third level is the more specific class and final level with all functional traits (46).

### Statistics analyses.

All statistical analyses were done in R v.4.1.1 (49). Compositional analyses were done in order to normalize sequence data using the centered log-ratio transformation test returned by the aldex.clr argument (ALDEx2 package version 1.21.1, [50]). A PCA was used to visualize (FactoMineR package version 2.3, [51]), and a perMANOVA analysis (adonis2 argument, Vegan package version 2.5–6, [52]) to determine the effect of agricultural practices (fertilizer application and tillage) and maize compartments (rhizosphere, roots and stem) on the bacterial community structure, functionality, and genes involved in the N cycle. The expected Benjamini-Hochberg corrected *P* value of the Welch´s *t* test of the bacterial groups, functionality, and genes involved in the N cycle was plotted versus the effect size in volcano plots (50). An effect size ≥0.8 and <1.3 or ≤−0.8 and <−1.3 was considered “large” and ≥1.3 or ≤−1.3 “very large” (53). The relative abundance of the most abundant bacterial phyla and species, functionality, and genes involved in the N cycle was given in barplots.

The taxonomic and functional diversity was determined with the Hill numbers at different *q* orders (*q *= 0, ^0^D_α_: species richness; q = 1, frequent species or ^1^D_α_: the exponential of Shannon entropy; and *q *= 2, dominant species or ^2^D_α_: the inverse of Simpson index) using the raw count data set at the species taxonomic level and all functions, respectively, as described by Ma and Li, (54) (2018) through the hillR package (55) with the argument hill_taxa.

### Data availability.

Sequences obtained in this study were submitted to NCBI database Sequence Read Archive (SRA) as biosamples under BioProject PRJNA818443 with the accession numbers SAMN26858625 to SAMN26858728.
