# Supplementary material for: Nitrogen Fertilizer Application Alters the Root Endophyte Bacterial Microbiome in Maize Plants, but Not in the Stem or Rhizosphere Soil
Source: Microbiol Spectr. 2022 Oct 18;10(6):e01785-22. doi: 10.1128/spectrum.01785-22 (PMC9769722; doi:10.1128/spectrum.01785-22)
Supplement: Supplemental file 1 — Supplemental material. Download spectrum.01785-22-s0001.pdf, PDF file, 1.7 MB [file spectrum.01785-22-s0001.pdf]

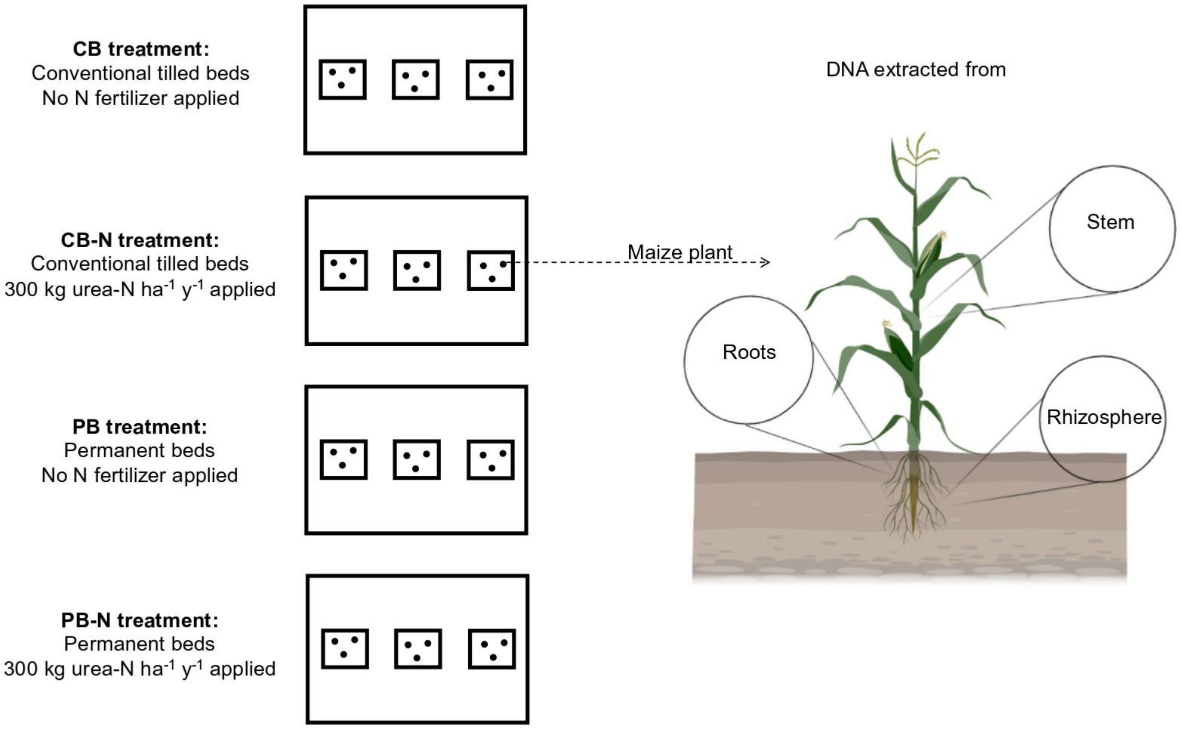

FIG S1 Sampling procedure and treatments sampled.

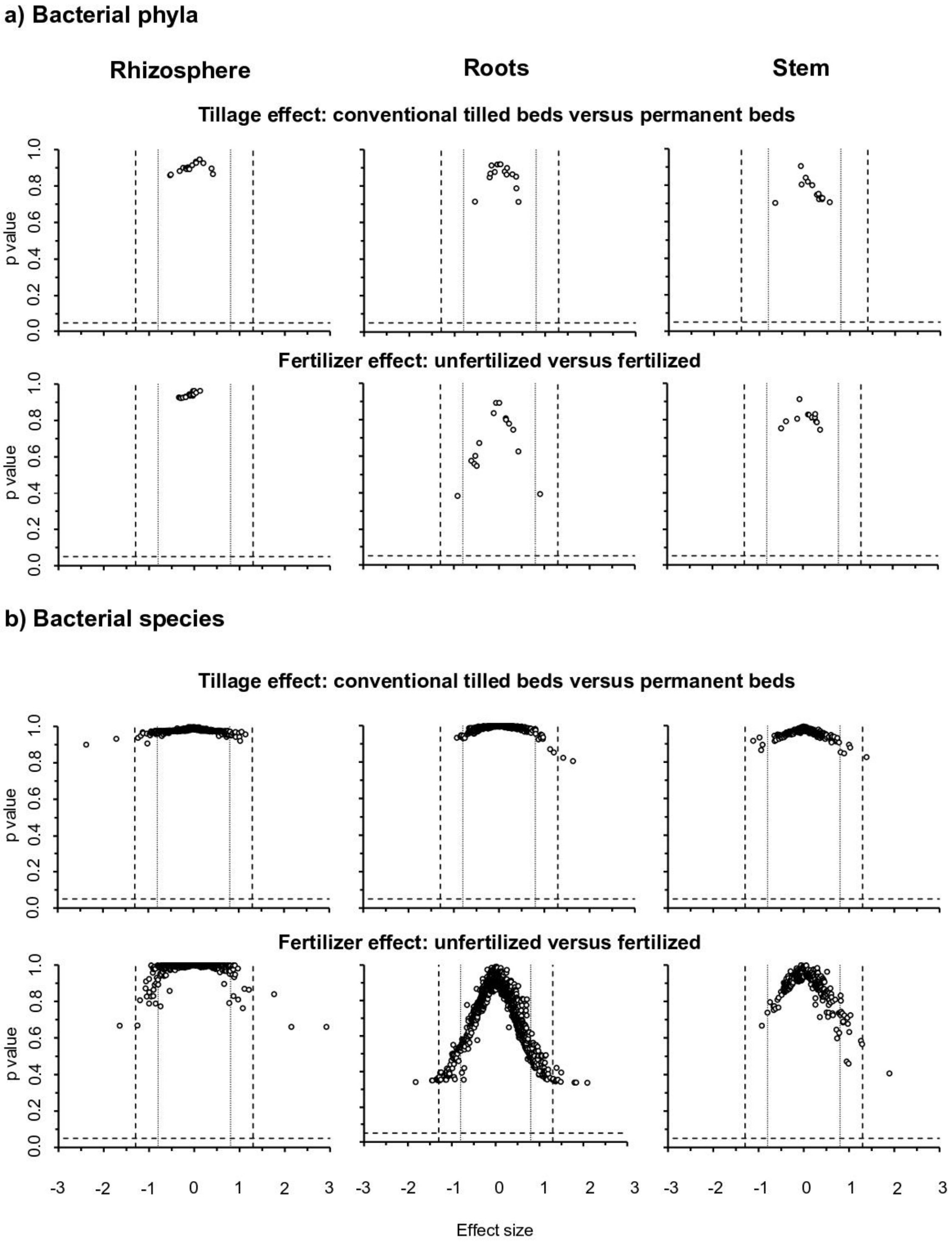

FIG S2 Volcano plot comparing the relative abundance of bacterial a) phyla and b) species in the fertilized versus the unfertilized, and tilled versus untilled soil in the rhizosphere, roots and stem of maize plants (*Zea mays* L.) with the effect size in the x-axis and the expected value of the Benjamini-Hochberg corrected p-value in the y-axis. The expected value of the Benjamini-Hochberg corrected p-value and the effect size, which is defined as the difference between groups divided by the maximum dispersion within group A or B, was calculated with the ALDEx2 package using the aldex.ttest argument (50).

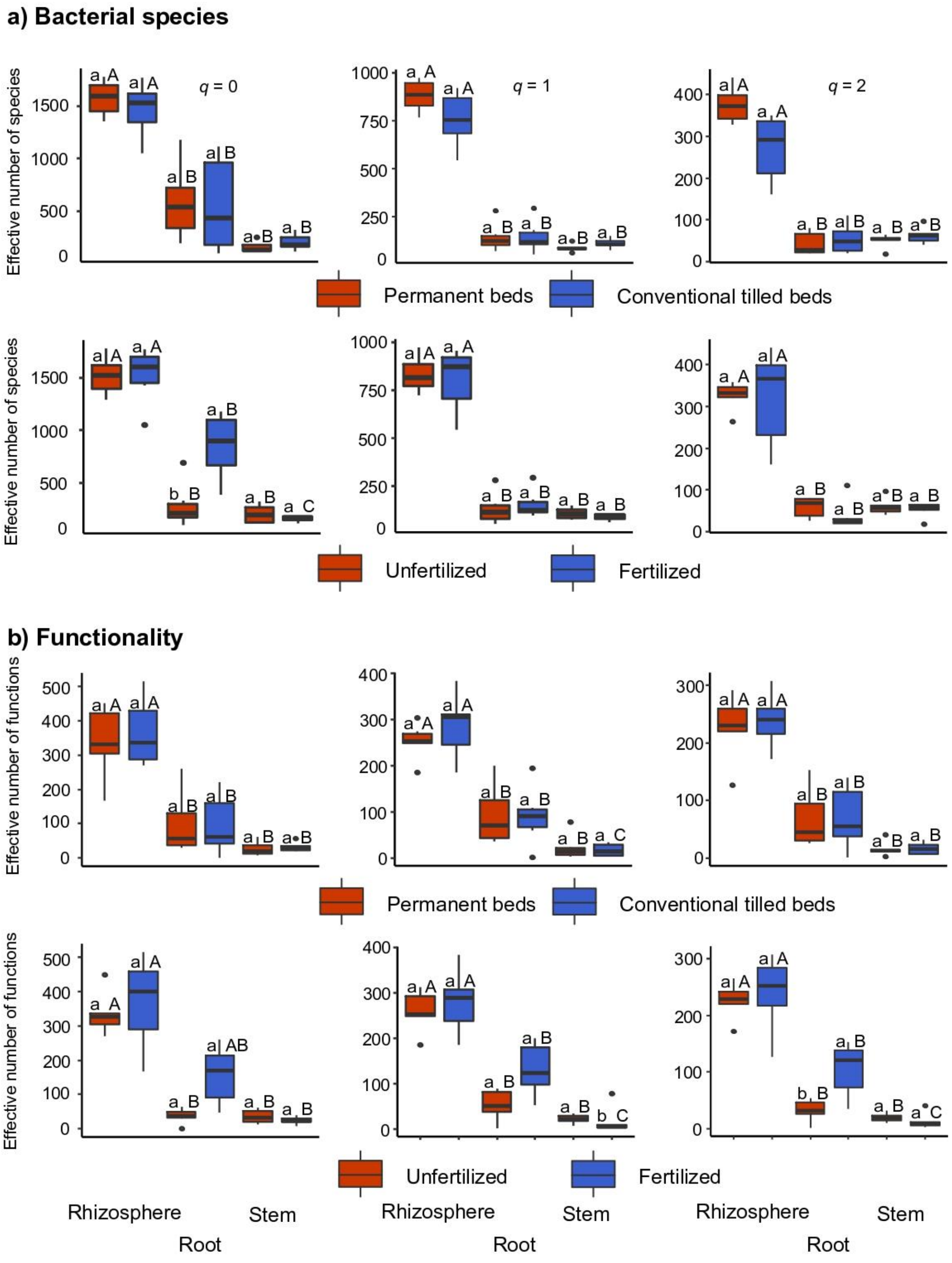

FIG S3 Effect of tillage, i.e permanent beds versus conventional beds and N fertilizer application, i.e. unfertilized versus fertilized, on Hill numbers of alpha taxonomic diversity of the a) bacterial species and b) functionality in the rhizosphere, roots and stem of maize (*Zea mays* L.). Values with the same capital letter are not significantly different between communities from different maize plant compartments under the same agricultural practices. Values with the same lowercase letters are not significantly different between communities from the same plant compartment of maize cultivated under different agricultural practices.

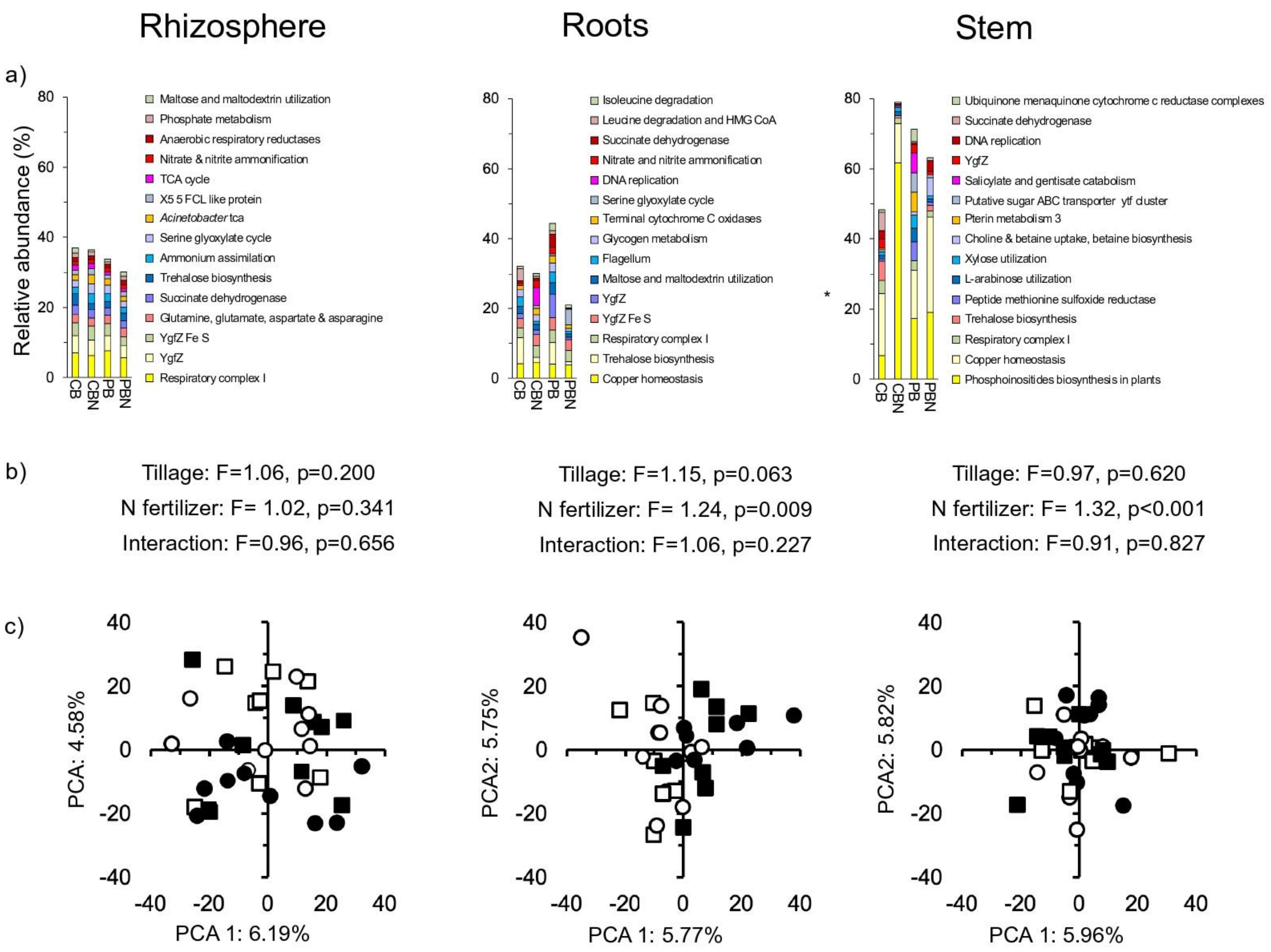

FIG S4 a) Relative abundance (%) of the most abundant bacterial traits at level 3 in the rhizosphere, roots and stem of maize (*Zea mays* L.) in soil with conventional tilled beds left unfertilized (CB) or fertilized (CB-N) and permanent beds left unfertilized (PB) or fertilized (PB-N), b) a permutational multivariate analyses of variance (perMANOVA) test to determine the effect of tillage, N fertilizer application and their interaction on the bacterial traits at level 3 and c) a principal component analysis (PCA) with all bacterial traits at level 3 in the CB (□), CB-N (■), PB (○) and PB-N (●).

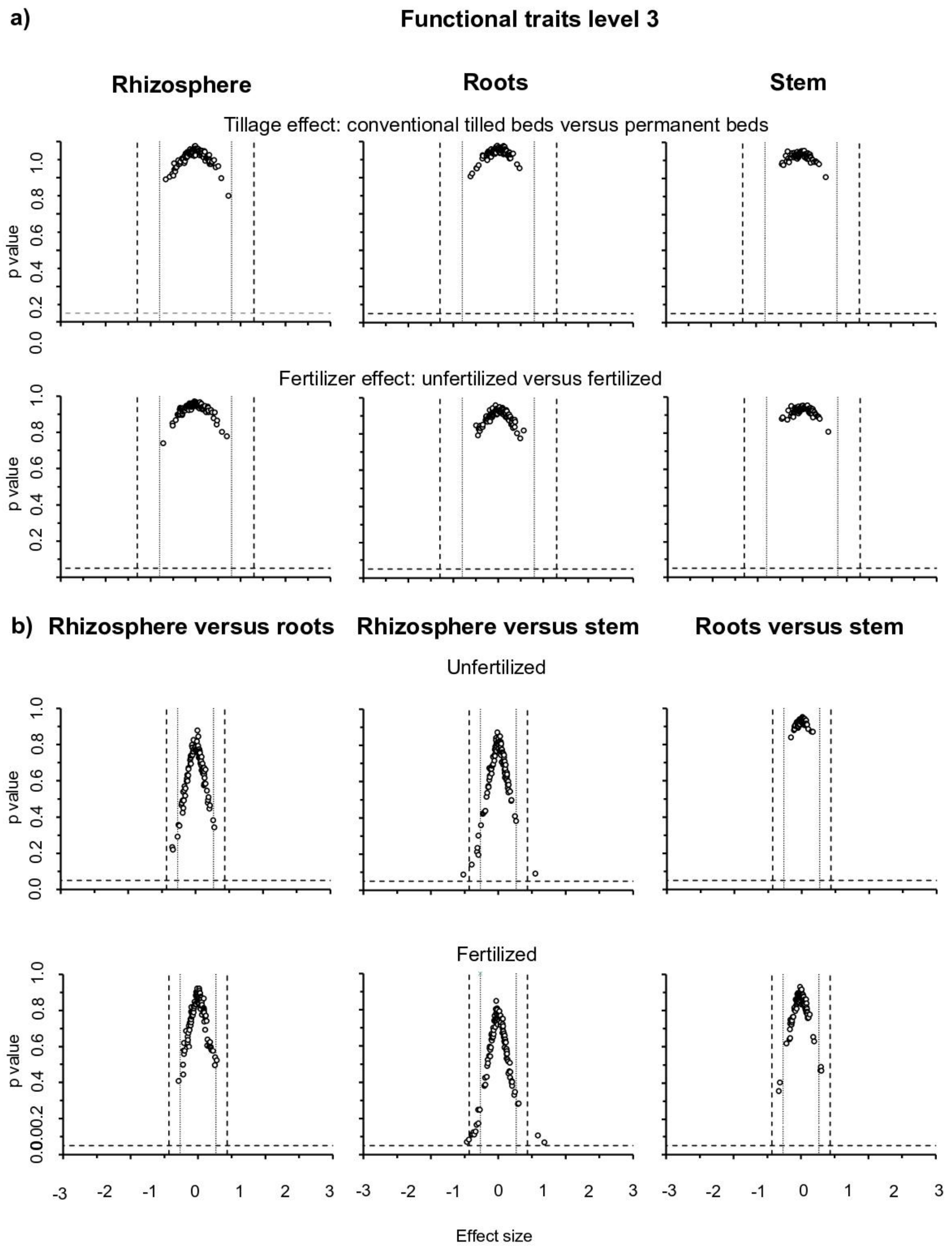

FIG S5 Volcano plot comparing the relative abundance of bacterial functional traits at level 3 in a) the fertilized versus the unfertilized, and tilled versus untilled soil in the rhizosphere, roots and stem of maize plants (*Zea mays* L.) and b) in the rhizosphere versus the roots or stem, and the roots versus the stem of maize plants in the unfertilized and fertilized soil with the effect size in the x-axis and the expected value of the Benjamini-Hochberg corrected p-value in the y-axis. The expected value of the Benjamini-Hochberg corrected p-value and the effect size, which is defined as the difference between groups divided by the maximum dispersion within group A or B, was calculated with the ALDEx2 package using the aldex.ttest argument (50).

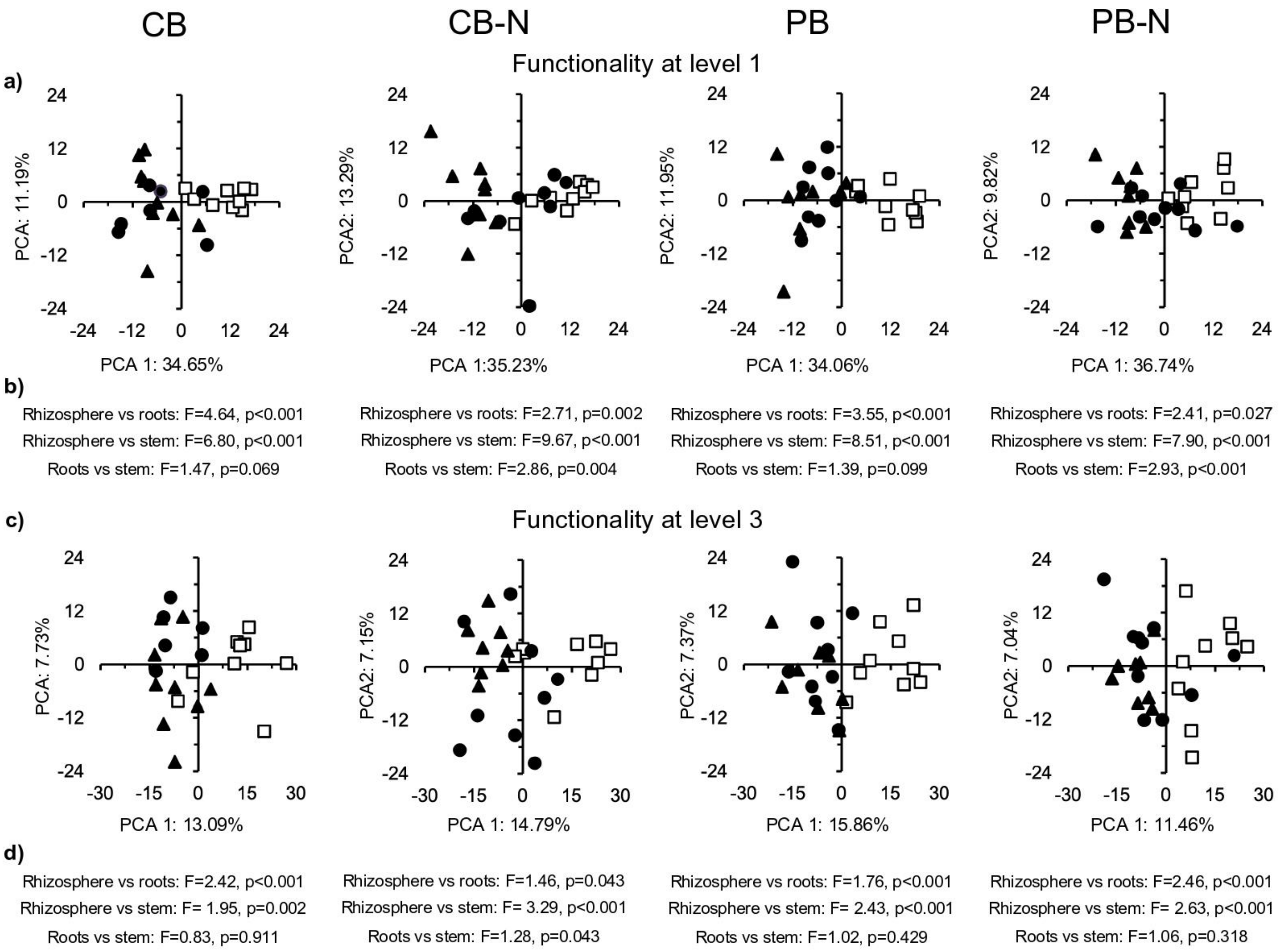

FIG S6 A principal component analysis (PCA) with a) the bacterial functional traits at level 1 in the rhizosphere (□), roots (●) and stem of maize (*Zea mays* L.) (▲) cultivated in soil with conventional tilled beds left unfertilized (CB) or fertilized (CB-N) and permanent beds left unfertilized (PB) or fertilized (PB-N), b) a permutational multivariate analyses of variance (perMANOVA) test comparing the bacterial community structure in the rhizosphere versus the roots and stem, and comparing the roots with the stem considering all bacterial phyla, c) a PCA with the bacterial functional traits at level 3 and d) a perMANOVA test comparing the bacterial functional traits at level 3 in the rhizosphere versus the roots and stem, and comparing the roots with the stem.

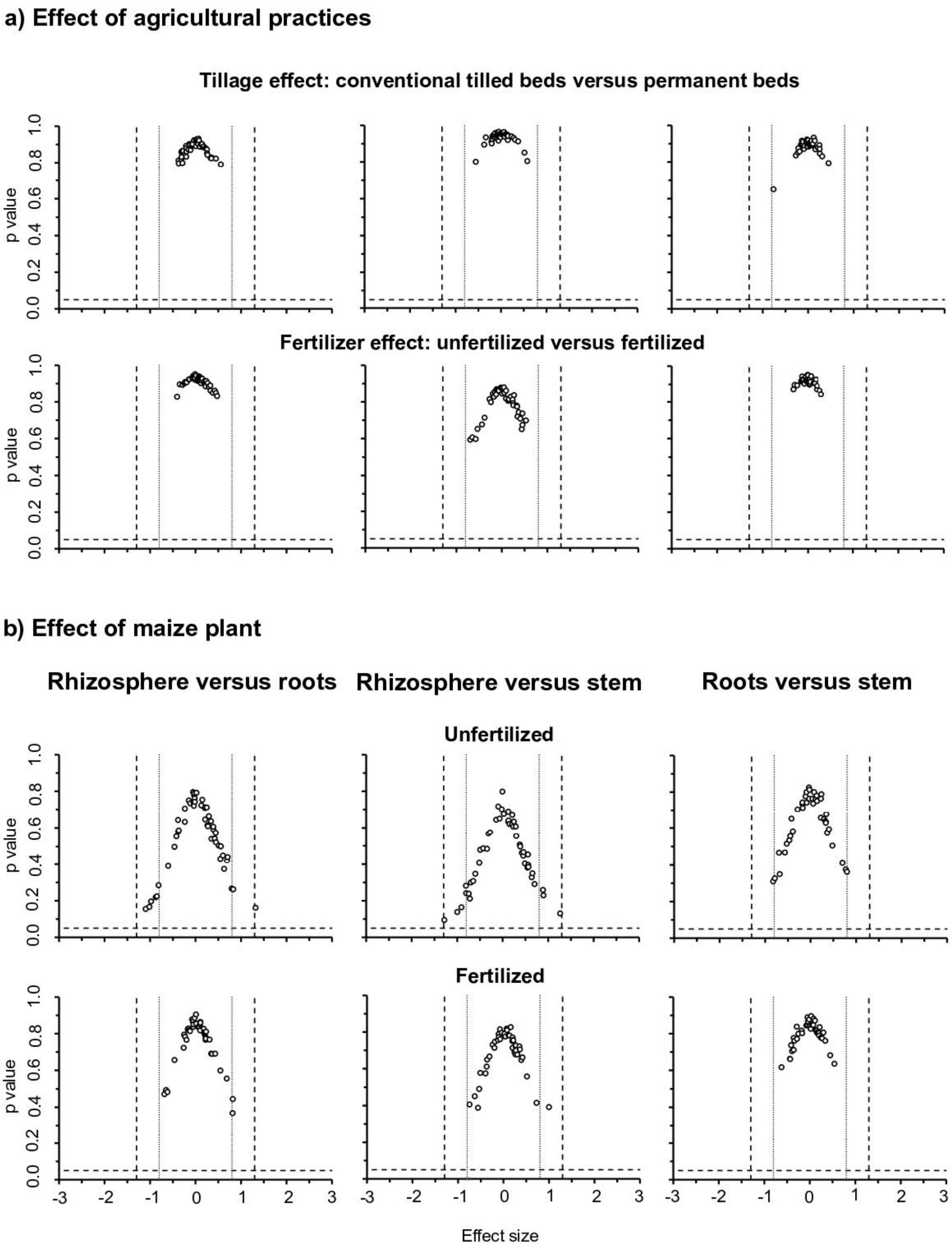

FIG S7 Volcano plot comparing the relative abundance of the genes involved in the N cycle in a) grouped per N cycling process or b) separate in the fertilized versus the unfertilized, and tilled versus untilled soil in the rhizosphere, roots and stem of maize plants (*Zea mays* L.) and b) in the rhizosphere versus the roots or stem, and the roots versus the stem of maize plants in the unfertilized and fertilized soil with the effect size in the x-axis and the expected value of the Benjamini-Hochberg corrected p-value in the y-axis (50).

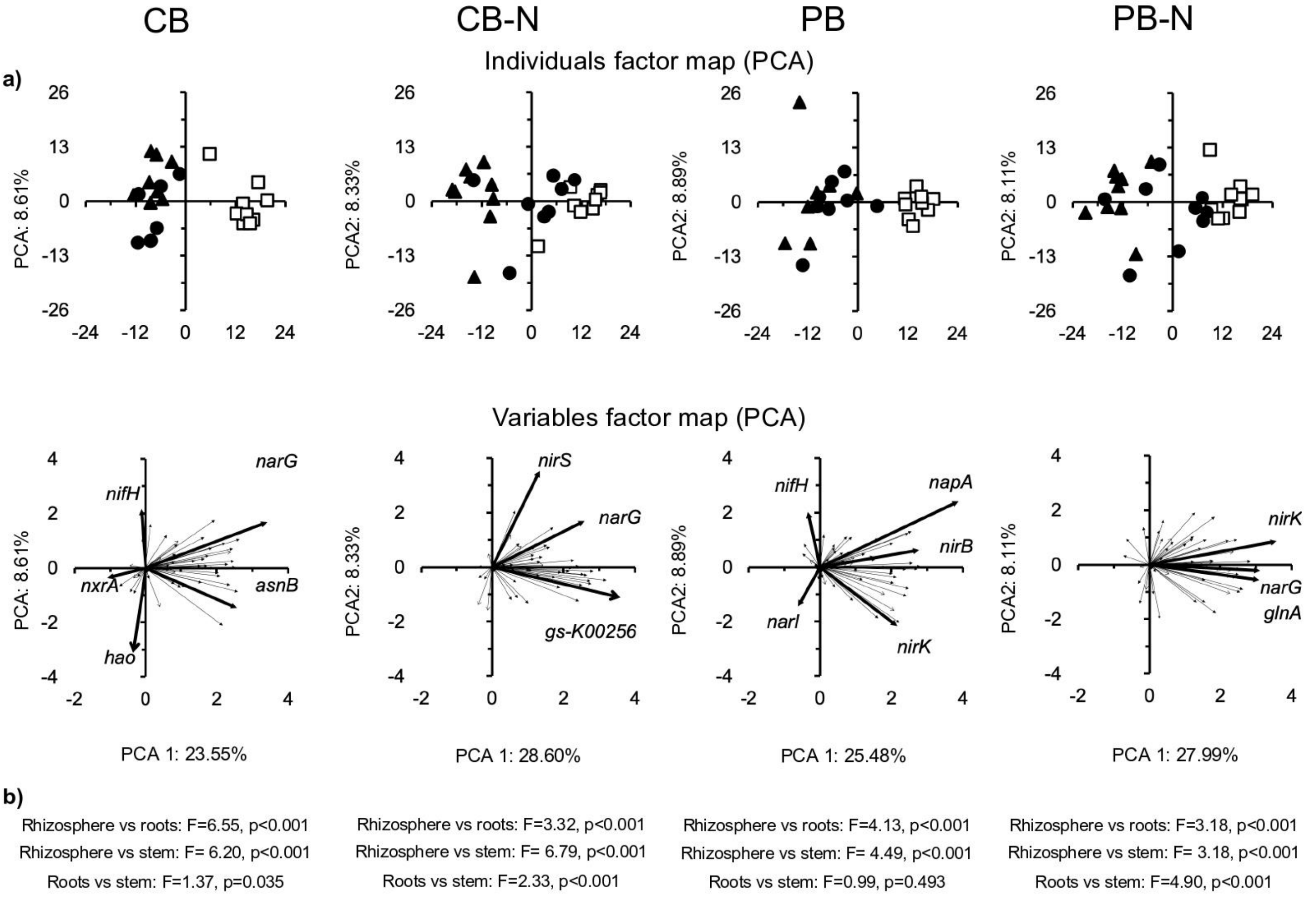

FIG S8 A principal component analysis (PCA) with a) the the genes involved in the N cycle in the rhizosphere (□), roots (●) and stem of maize (*Zea mays* L.) (▲) cultivated in soil with conventional tilled beds left unfertilized (CB) or fertilized (CB-N) and permanent beds left unfertilized (PB) or fertilized (PB-N), b) a permutational multivariate analyses of variance (perMANOVA) test comparing the bacterial community structure in the rhizosphere versus the roots and stem, and comparing the roots with the stem considering the genes involved in the N cycle.

Table S1. Number of sequences and taxon and functional traits represented in samples of each treatment of stem, root, and rhizosphere of maize plants (*Zea mays* L.).

		Stem				Root				Rhizosphere				Total
		CB [a]	CB-N [b]	PB [c]	PB-N [d]	CB	CB-N	PB	PB-N	CB	CB-N	PB	PB-N	
		Number of sequences												
		6877027	8299628	7744499	8474756	16379340	12824988	12398770	12255004	12989880	11795476	13172393	12582623	135794384
Bacterial taxonomy	Phyla	13	11	12	11	12	13	13	14	16	17	17	17	18
	Genera	214	148	157	140	200	481	355	447	646	651	656	656	760
	Species	389	237	268	210	294	1423	806	1329	1796	1862	1855	1892	2388
Microbiome functionality [e]	Subsystem 1	23	20	20	18	22	29	19	25	29	31	27	29	31
	Subsystem 2	40	31	34	28	31	56	35	63	73	88	80	79	104
	Subsystem 3	77	56	65	49	78	202	83	222	323	381	320	320	532
	All functions	106	67	81	66	90	391	113	386	666	859	731	705	1824

[a] CB: conventional tilled beds left unfertilized, [b] CB-N conventional tilled beds fertilized, [c] PB: permanent beds left unfertilized, [d] PB-N: permanent beds fertilized, [e]The microbial functionality was organized by subsystem structure the first level is the most general class and the third level is the more specific class, the final level involved all functionality functions (all functions) (47).

Table S2. The bacterial groups in the rhizosphere (Rhi), roots or stem as affected by agricultural practice (conventional beds (CB) versus permanent beds (PB)) or fertilizer application rate (no fertilizer applied to soil (F0) or soil fertilized with 300 kg urea-N ha^{-1} (F300) using a compositional approach, i.e., analysis of differential abundance taking sample variation into account (ALDEx2 package, version 1.21.1, date 2020-04-20, 50).

Effect of agricultural practice, i.e. permanent beds or conventional beds, on the bacterial groups

		Relative abundance (%)				
		CB	CB	PB	PB	
Rhizosphere		Rhi	Roots	Rhi	Roots	Effect size
Pontibacter pudoricolor	CB>PB	0.01	0.00	0.00	0.00	2.7
Enterobacter hormaechei	CB>PB	0.14	0.13	0.00	0.22	1.7
Roots						
Conexibacter woesei	PB>CB	0.21	0.02	0.27	2.13	1.4
Mycolicibacterium tokaiense	PB>CB	0.08	0.01	0.14	0.16	1.6
Stem			Stem		Stem	
Rhodococcus sp PBTS-1	PB>CB	0.04	0.29	0.05	0.54	1.4

Effect of fertilizer application, i.e. 0 or 300 kg urea-N ha^{-1}, on the bacterial groups

		F0	F0	F300	F300	
Rhizosphere		Rhi	Roots	Rhi	Roots	Effect size
Streptomyces sp Z022	F0>F300	0.03	0.00	0.00	0.00	1.7
Nitrosomonas	F300>F0	0.00	0.00	0.04	0.00	3.3
Nitrosomonas communis	F300>F0	0.00	0.00	0.03	0.00	2.9
Nitrosospira	F300>F0	0.01	0.00	0.06	0.00	1.4
Nitrosospira multiformis	F300>F0	0.00	0.00	0.05	0.00	2.1
Sphingobium japonicum	F300>F0	0.00	0.08	0.06	0.03	1.8
Roots						
Achromobacter denitrificans	F300>F0	0.08	0.00	0.07	0.07	2.1
Bosea vaviloviae	F300>F0	0.07	0.01	0.06	0.07	1.5
Bradyrhizobium sp 6 2017	F300>F0	0.07	0.00	0.07	0.06	1.8
Cellulomonas fimi	F300>F0	0.11	0.00	0.09	0.93	1.5
Enterobacter asburiae	F300>F0	0.09	0.00	0.34	0.45	1.4
Hydrogenophaga sp BA0156	F300>F0	0.23	0.02	0.23	0.08	1.4
Mesorhizobium terrae	F300>F0	0.05	0.00	0.05	0.03	1.4
Microbacterium testaceum	F300>F0	0.04	0.00	0.04	0.02	1.3
Stenotrophomonas	F300>F0	1.08	1.88	1.29	6.30	0.9
Stenotrophomonas acidaminiphila	F300>F0	0.12	0.07	0.19	0.36	1.4
Stenotrophomonas rhizophila	F300>F0	0.07	0.04	0.09	0.15	1.4
Stenotrophomonas sp SAU14A_NAIMI4_5	F300>F0	0.01	0.00	0.01	0.08	1.8
Thermomonas sp HDW16	F300>F0	0.03	0.00	0.05	0.03	1.3
Burkholderia ambifaria	F0>F300	0.02	1.50	0.03	0.20	1.3
Cupriavidus metallidurans	F0>F300	0.07	0.53	0.07	0.04	1.3
Janthinobacterium.sp.17J80.10	F0>F300	0.02	0.90	0.02	0.12	1.3
Kitasatospora setae	F0>F300	0.03	0.86	0.04	0.11	1.4
Mameliella alba	F0>F300	0.01	0.47	0.01	0.05	1.4
Myxococcus xanthus	F0>F300	0.14	0.90	0.13	0.12	1.3
Rhodococcus opacus	F0>F300	0.17	0.53	0.07	0.04	1.3
Streptomyces sp. SCUT3	F0>F300	0.05	0.99	0.01	0.05	1.3
Stem			Stem		Stem	
Mycobacterium intracellulare 1.9)	F300>F0	0.02	0.57	0.01	0.68	1.9

* The size effect was calculated with the ALDEx2 package using the aldex.ttest which calculate Wilcoxon Rank Sum test and Welch's t-test statistics and is defined as the difference between groups divided by the maximum dispersion within group A or B. Only bacterial species with a size effect that was considered “very large” > 1.3 as reported by Kim (53) are given.

Table S3. The bacterial phyla as affected by cultivation of maize plants (*Zea mays* L.) (i.e., comparing the rhizosphere with the roots and stem, and the roots with the stem) using a compositional approach, i.e. analysis of differential abundance taking sample variation into account (ALDEx2 package, 50).

Bacterial groups in the rhizosphere (rhi) versus those in the roots (roo) of maize plants (*Zea mays* L.)	
Unfertilized soil	Fertilized with 300 kg urea-N ha^{-1}
Chlorobi (rhi > roo, 1.87 *, p=0.009 **),<br>Nitrospirae (rhi > roo, 1.54, p=0.020),<br><br>Actinobacteria (roo > rhi, 2.68, p=0.006),<br>Cyanobacteria (roo > rhi, 1.78, p=0.016),<br>Firmicutes (roo > rhi, 1.51, p=0.040),<br>Proteobacteria (roo > rhi, 2.66, p=0.007),<br>Verrucomicrobia (roo > rhi, 3.36, p=0.006)	Acidobacteria (rhi > roo, 3.05, p=0.005),<br>Chlorobi (rhi > roo, 1.38, p=0.027),<br><br>Actinobacteria (roo > rhi, 3.09, p=0.005),<br>Cyanobacteria (roo > rhi, 3.53, p=0.005),<br>Firmicutes (roo > rhi, 1.75, p=0.011),<br>Gemmatimonadetes (roo > rhi, 1.45, p=0.026),<br>Proteobacteria (roo > rhi, 1.56, p=0.018),<br>Tenericutes (roo > rhi, 1.36, p=0.036),<br>Verrucomicrobia (roo > rhi, 2.87, p=0.005)
Bacterial groups in the rhizosphere (rhi) versus those in the stem of maize plants (*Zea mays* L.)	
Nitrospirae (rhi > stem, 1.87, p=0.009),<br>Chlorobi (rhi > stem, 1.54, p=0.020),<br><br>Actinobacteria (stem > rhi, 2.68, p=0.006),<br>Cyanobacteria (stem > rhi, 1.78, p=0.016),<br>Firmicutes (stem > rhi, 1.51, p=0.040),<br>Proteobacteria (stem > rhi, 2.66, p=0.007),<br>Verrucomicrobia (stem > rhi, 3.36, p=0.006)	Acidobacteria (rhi > stem, 3.02, p=0.004),<br>Nitrospirae (rhi > stem, 1.59, p=0.014),<br><br>Actinobacteria (stem > rhi, 4.38, p=0.004),<br>Cyanobacteria (stem > rhi, 5.26, p=0.004),<br>Firmicutes (stem > rhi, 2.41, p=0.005),<br>Gemmatimonadetes (stem > rhi, 2.23, p=0.006),<br>Planctomycetes (stem > rhi, 1.84, p=0.008),<br>Proteobacteria (stem > rhi, 2.02, p=0.005),<br>Verrucomicrobia (stem > rhi, 4.41, p=0.004)
Bacterial groups in the roots (roo) versus those in the stem of maize plants (*Zea mays* L.)	
	Acidobacteria (roo > stem, 2.50, p=0.016),<br><br>Cyanobacteria (stem > roo, 2.31, p=0.019)

* The size effect was calculated with the ALDEx2 package using the aldex.ttest which calculate Wilcoxon Rank Sum test and Welch's t-test statistics and is defined as the difference between groups divided by the maximum dispersion within group A or B. Only bacterial species with a size effect that was considered “very large” > 1.3 as reported by Kim (53) are given,
** The corresponding expected value of the Benjamini-Hochberg corrected p-value for each feature.

Table S4. The bacterial species as affected by cultivation of maize plants (*Zea mays* L.) (i.e. comparing the rhizosphere with the roots and stem, and the roots with the stem) using a compositional approach, i.e. analysis of differential abundance taking sample variation into account (ALDEx2 package, 50).

Unfertilized soil	Fertilized with 300 kg urea-N ha^{-1}
Bacterial groups more abundant in the rhizosphere than in the roots of maize plants	
Magnetospirillum gryphiswaldense (3.41*, p=0.022**),<br>*Streptomyces hygroscopicus* (2.20, p=0.020),<br>*Minicystis rosea* (2.03, p=0.024),<br>*Burkholderia pseudomallei* (2.01, p=0.022),<br>*Bradyrhizobium lablabi* (2.00, p=0.023)	*Myxococcus stipitatus* (2.43, p=0.028),<br>*Thauera humireducens* (2.40, p=0.028),<br>*Arthrobacter sp QXT.31* (2.37, p=0.028),<br>*Micromonospora viridifaciens* (2.27, p=0.028),<br>*Denitratisoma sp DHT3* (2.25, p=0.028)
Bacterial groups more abundant in the roots of maize plants than in the rhizosphere	
Roseimicrobium sp ORNL1 (8.49, p=0.022),<br>*Azospirillum sp TSH100* (7.11, p=0.022),<br>*Micromonospora sagamiensis* (7.01, p=0.040),<br>*Sphingomonas paucimobilis* (6.76, p=0.022),<br>*Streptomyces sp SCUT.3* (6.28, p=0.022)	*Cellulosimicrobium sp TH 20* (4.21, p=0.027),<br>*Streptomyces bacillaris* (3.65, p=0.027)<br>*Bosea sp F3.2* (3.62, p=0.027),<br>*Bosea sp ANAM02* (3.53, p=0.027),<br>*Azospirillum sp TSH100* (3.48, p=0.027)
Bacterial groups more abundant in the rhizosphere than in the stem of maize plants	
Magnetospirillum gryphiswaldense (3.40, p=0.013),<br>*Lentzea guizhouensis* (2.67, p=0.013),<br>*Luteitalea pratensis* (2.35, p=0.013),<br>*Rhodoplanes sp Z2 YC6860i* (2.24, p=0.013),<br>*Bradyrhizobium diazoefficiens* (2.23, p=0.013)	*Magnetospirillum gryphiswaldense* (3.10, p=0.012),<br>*Variovorax paradoxus* (2.77, p=0.012),<br>*Lentzea guizhouensis* (2.73, p=0.012),<br>*Ramlibacter tataouinensis* (2.68, p=0.012),<br>*Rubrivivax gelatinosus* (2.38, p=0.012)
Bacterial groups more abundant in the stem of maize plants than in the rhizosphere	
Pandoraea vervacti (13.65, p=0.013),<br>*Roseimicrobium sp ORNL1* (9.61, p=0.013),<br>*Nocardia wallacei* (9.54, p=0.013),<br>*Micromonospora sagamiensis* (9.51, p=0.013),<br>*Isoptericola variabilis* (8.99, p=0.013)	*Janthinobacterium sp 17J80 10* (19.21, p=0.012),<br>*Streptomyces sp SCUT 3* (14.71, p=0.012),<br>*Sphingomonas paucimobilis* (12.71, p=0.012),<br>*Massilia armeniaca* (12.12, p=0.012),<br>*Micromonospora sagamiensis* (11.12, p=0.012)
Bacterial groups more abundant in the roots of maize plants than in the stem	
Lentzea guizhouensis (2.77, p=0.117),<br>*Bradyrhizobium sp CCBAU 51765* (1.74, p=0.243),<br>*Bradyrhizobium diazoefficiens* (1.65, p=0.220),<br>*Sinorhizobium fredii* (1.65, p=0.218),<br>*Bradyrhizobium icense* (1.35, p=0.230)	*Bradyrhizobium diazoefficiens* (3.39, p=0.026),<br>*Lentzea guizhouensis* (3.21, p=0.026),<br>*Rhizobium leguminosarum* (2.80, p=0.026),<br>*Agrobacterium tumefaciens* (2.74, p=0.026),<br>*Bradyrhizobium sp CCBAU 51765* (2.71, p=0.026)
Bacterial groups more abundant in the stem of maize plants than in the roots	
Aeromicrobium erythreum (3.23, p=0. 117),<br>*Rhodococcus sp PBTS 1* (3.13, p=0.117),<br>*Gordonia polyisoprenivorans* (2.71, p=0. 117),<br>*Streptomyces hygroscopicus* (2.63, p=0. 117),<br>*Gordonia terrae* (2.08, p=0.117)	*Pseudomonas sp VLB120* (3.20, p=0.026),<br>*Gordonia polyisoprenivorans* (2.96, p=0.026),<br>*Cutibacterium acnes* (2.74, p=0.026),<br>*Pseudonocardia sp AL041005 10* (2.72, p=0.026),<br>*Lichenihabitans psoromatis* (2.69, p=0.026)

* The size effect was calculated with the ALDEx2 package using the aldex.ttest which calculate Wilcoxon Rank Sum test and Welch's t-test statistics and is defined as the difference between groups divided by the maximum dispersion within group A or B. Only the five bacterial species with the largest size effect and considered “very large” > 1.3 as reported by Kim (53) are given, ** The corresponding expected value of the Benjamini-Hochberg corrected p-value for each feature.
